# Knowledge, Attitudes and Practices Regarding Sylvatic Rabies among High-Risk Households in Ceará State, Brazil

**DOI:** 10.3390/tropicalmed6040209

**Published:** 2021-12-08

**Authors:** Naylê Francelino Holanda Duarte, Patrícia Pereira Lima Barbosa, Danielle Bastos Araujo, Silvana Regina Favoretto, Phyllis Catharina Romijn, Raphael William Pontes Neres, Raquel Holanda Varela, Walber Feijó de Oliveira, Carlos Henrique Alencar, Jorg Heukelbach

**Affiliations:** 1 Postgraduate Program in Public Health, School of Medicine, Federal University of Ceará, Fortaleza 60430-140, CE, Brazil; nayle.holanda@gmail.com (N.F.H.D.); patricialima18@yahoo.com.br (P.P.L.B.); carllosalencar@ufc.br (C.H.A.); 2 Institute of Biomedical Sciences, University of São Paulo, São Paulo 05508-000, SP, Brazil; daniellebastos@yahoo.com.br (D.B.A.); srfavoretto@usp.br (S.R.F.); 3 Agricultural Research Institute of Rio de Janeiro, Rio de Janeiro 24120-191, RJ, Brazil; phyllisromijn@gmail.com; 4 School of Veterinary Medicine, Ceará State University, Fortaleza 60714-903, CE, Brazil; neresraphael@gmail.com; 5 Department of Biology, Federal University of Ceará, Fortaleza 60440-900, CE, Brazil; raquelhvarela@gmail.com; 6 Brazilian Institute of Environment and Renewable Natural Resources, Wild Animal Sorting Center, Fortaleza 60843-150, CE, Brazil; walberfeijo@yahoo.com.br

**Keywords:** rabies, animals, wild, raising domestic animals, capuchin monkey, marmoset, Brazil

## Abstract

Rabies transmitted by sylvatic populations has become an increasing concern in Brazil. A total of 113 participants with a history of contact with sylvatic populations were interviewed in 27 municipalities of Ceará State in northeast Brazil. Questionnaires included questions on knowledge, attitudes and practices (KAP) regarding sylvatic rabies. Most of the respondents (92%) knew about rabies and confirmed at least one species that transmitted the disease (79.6%). Of these respondents, 69% mentioned monkeys, and 67.2% mentioned dogs. However, 16% of the respondents listed an incorrect species. In general, knowledge on the symptoms and signs and on prevention measures was weak. The majority raised pets (93.8%), most commonly dogs and cats, and, of all the pets, 85.7% were claimed to be vaccinated against rabies. A total of 67.3% reported the appearance of free-living wild animals around their houses, mostly marmosets and wild canids; 18.3% reported that sylvatic populations had attacked animals or humans. Seventy-three percent had raised or still were raising wild animals as pets, mostly capuchin monkeys (79.5%) and marmosets (24.1%). This is the first KAP study on sylvatic rabies in Brazil. The data indicate important knowledge gaps and risk behavior within a high-risk population. There is a need for strengthening and improving sylvatic rabies surveillance and control, combined with the intensification of education and information campaigns.

## 1. Introduction

Rabies is a neglected tropical disease of major global importance, with thousands of deaths per year worldwide. About 95% of the deaths occur in Africa and Asia. Worldwide, domestic dogs are responsible for transmission in 99% of the cases [1,2].

Similar to most other infectious diseases, low socio-economic strata and rural areas are considered to be at the highest risk [3,4]. In addition to the lack of knowledge regarding the risk of acquiring the disease after exposure, populations in rural areas suffer from limited access to the healthcare system, and financial constraints may hinder travel to the local hospital for post-exposure prophylaxis [4].

The transmission cycle of the rabies virus maintained by dogs and cats can be controlled through effective prophylactic measures [2]. However, the emergence of transmission by sylvatic populations has become an increasing challenge, especially in Brazil [5,6]. In a rural community in the Brazilian State of Amazonas, 57% of the respondents had informal knowledge about rabies, but only 23% had received information from health professionals [7]. In a Pakistani study, all the respondents had heard about the disease and knew about the importance of domestic animals for transmission, especially dogs, which were mentioned by 93% of the respondents [8]. On the other side, only 22% identified cats as putative transmitters [8]. Knowledge regarding the wildlife species involved in the rabies virus transmission was limited in an Indian study where foxes and monkeys were cited as possible transmitters by only about 25% of the participants [9]. In rural Ethiopia, people believed that traditional healers and herbal medicines may cure rabies, indicating the importance of health education in rabies prevention measures, sensitively addressing cultural and gender concerns [10].

We have recently shown that the rabies virus in the State of Ceará in northeast Brazil is transmitted mainly by wild animals [5,6]. The last outbreak of rabies transmitted by dogs in Ceará occurred in 2003 [11]. Of the last six cases of human rabies since 2006, five were transmitted by wild animals [12]. Surveillance and control measures in the state should, therefore, focus on rabies caused by wildlife [12]. However, the population apparently lacked basic knowledge about the disease and the transmission cycle involving sylvatic populations. Thus, knowledge, attitudes and practices (KAP) were studied among people who had contact with or raised wild mammals as companion animals in the State of Ceará.

## 2. Materials and Methods

### 2.1. Study Area and Population

**Figure 1:**
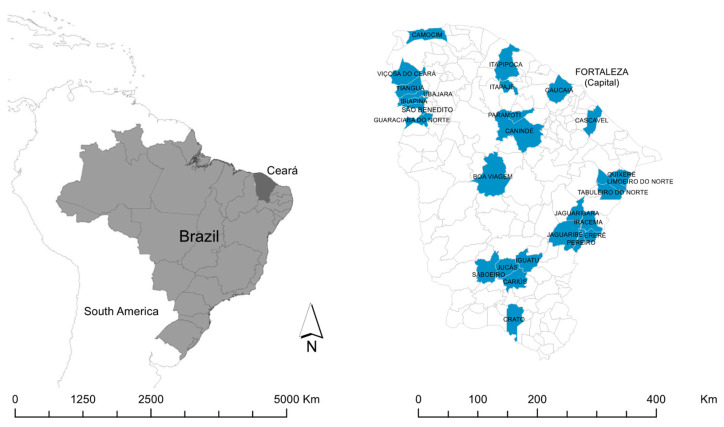
Location of Ceará State in Brazil, and location of the municipalities included in the study.

The State of Ceará in the northeast region of Brazil is bordered in the north by the Atlantic Ocean, in the west by the State of Piauí, in the east by the States of Rio Grande do Norte and Paraíba, and in the south by the State of Pernambuco (Figure 1). Ceará has a population of approximately nine million and an area of 149 thousand km^2^, distributed throughout 184 municipalities [13].

In Ceará, rabies virus transmission dynamics have changed during the last few decades, and rabies transmitted by dogs is now controlled. Almost all cases in humans that occurred during the last 18 years were transmitted by wild animals [12].

We performed a KAP study in 27 municipalities dispersed throughout the state (Figure 1). The selection of municipalities was carried out in collaboration with local health professionals (endemic control agents and community health agents) who conducted an active survey of municipalities with records of people with a history of contact with an aggressive wild animal, captive breeding of wild animals and/or interaction of household members with free-living wild animals. These high-risk households were identified in cooperation with community agents. Participants were included and invited for an interview if ≥18 years of age and in the presence of one or both of the following characteristics: the household member has had contact with animals kept in captivity or with free-living animals; history of any aggression by wild mammals.

### 2.2. Variables and Data Collection

Endemic control agents (agentes de controle de endemias—ACE in Portuguese) and community health agents (agentes comunitários de saúde—ACS in Portuguese) conducted the surveys in their respective municipalities. Participants were interviewed using pre-tested structured questionnaires. The questionnaires consisted of five blocks of closed and open questions. Variables included socio-demographic data, and questions on knowledge, attitudes and practices regarding rabies and its control, with focus on wildlife-mediated transmission (Table 1).

We asked for different aspects of knowledge on rabies, risk behaviors, exposures and attitudes and behaviors in relation to capturing and raising wild animals. Feral dogs were included in the group of domestic dogs.

In the first 34 questionnaires applied, the variable regarding the knowledge of people who might have been attacked by mammals was not included. Only after some people mentioned these incidents did we insert this information as a variable.

During the visits, the team was introduced to the family, and the investigator identified the person with most intensive contact with the animals to answer the questionnaire by reading out the questions. If this specific person was less than 18 years old, a parent or guardian was asked to answer the questions. After application of questionnaires, the team in charge conducted education on environmental legislation, risk of zoonotic transmission and animal welfare.

### 2.3. Statistical Analysis

A database was created to consolidate and organize the data using MS Excel 2016 software (Microsoft Corporation, Redmond, WA, USA). Analysis was performed using Stata software version 15.1 (StataCorp, College Station, TX, USA). Categorical variables were presented in absolute and relative frequencies. In the case of numerical variables, measures of central tendency and dispersion were chosen according to normality criteria: mean and standard deviation for normally distributed variables and median and interquartile range for non-normal variables. Answers to open questions were categorized and grouped. Knowledge was stratified according to area of residence (rural/urban).

### 2.4. Ethical Aspects

The study was approved by the Ethical Review Boards of the Federal University of Ceará (CAAE number—13466719.6.0000.5054) and of the State Health Department (CAAE number—13466719.3001.5051). The municipalities gave their consent for the study. Before application of the questionnaires, the objectives of the study were explained to the participants and that participation would be on a voluntary basis. Informed written consent was obtained. Personal data were kept strictly confidential.

## 3. Results

### 3.1. Characteristics of Study Population

A total of 113 participants were included in the study; there were no refusals to participate in the study. The median number of inhabitants per residence was three (interquartile range: 2–4). Most respondents were females, had elementary school education and resided in rural areas (Table 2).

### 3.2. Knowledge

In total, 92% of the respondents—97% in rural areas and 87% in urban areas—said that they had heard of rabies, and that they knew at least one animal species that can transmit the disease (Table 3).

**Figure 2:**
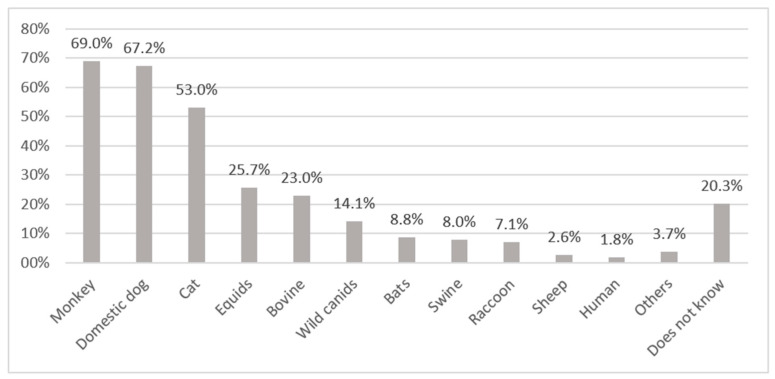
Animals or categories of animals reported by respondents to be rabies virus transmitting animals. Others: snake (0.9%), parrot (0.9%), jaguar (0.9%), goat (0.9%), opossum (0.1%).

In general, knowledge of the animal species that may transmit rabies was low. Of the 80% who reported knowing the transmission species, 16% reported incorrect species. About 67–69% mentioned correctly domestic dogs and monkeys, and 53% cats, while about 20% were not able to respond to this question (Figure 2). As domestic dogs also roam freely around in the communities, the respondents did not differentiate between feral and domestic dogs.

**Figure 3:**
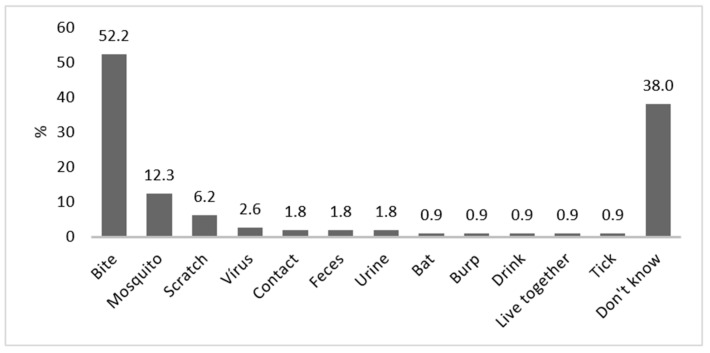
Modes of transmission for rabies, as reported by respondents (bites, scratches and bats were considered as correct answers). Contact means touching and/or feeding the animals; live together means that animals are leashed or kept in cages on the property.

Knowledge on the mode of transmission was weak, and 38% did not know the mode of transmission at all. About 52% mentioned correctly animal bites and 6% scratches, but 12% believed that mosquitoes would transmit rabies. Other incorrect modes of transmission cited by respondents included transmission by urine and feces (Figure 3).

**Figure 4:**
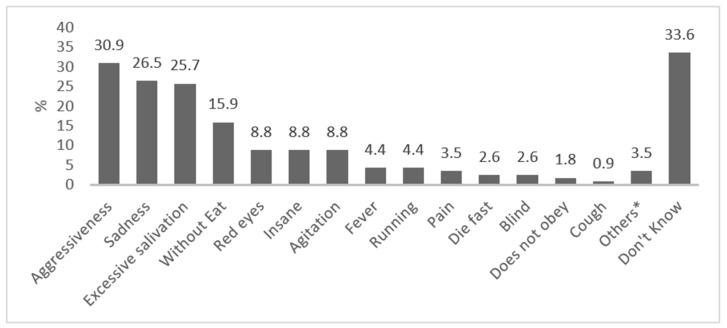
Symptoms of rabies, as reported by respondents. * Others: barking (0.9%), not drinking sufficiently (0.9%), stiff tail (0.9%), quivering (0.9%).

The symptoms/clinical signs cited by the respondents are presented in Figure 4. Three common symptoms and signs—aggressiveness, salivation and anorexia—were among the topmost mentioned responses.

**Figure 5:**
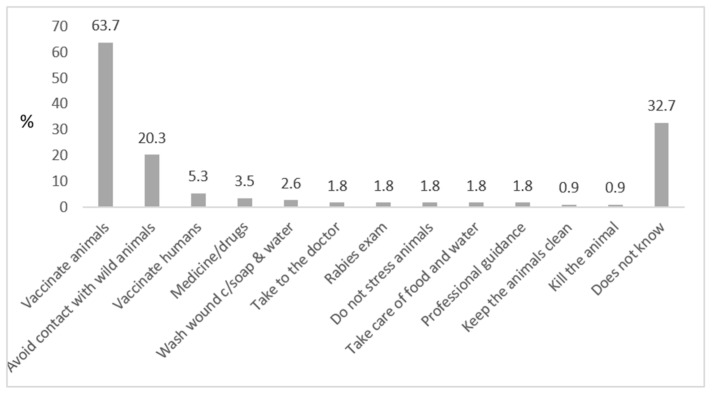
Knowledge on prevention of rabies, as reported by respondents (vaccination of animals and avoiding contact with wild animals were considered as correct answers). Killing the animal was considered an incorrect answer; after an act of aggression, domestic animals (esp. dogs) should be observed for a defined period at a safe place (without the possibility of contact to other animals or to humans).

Almost 64% answered animal vaccination as a form of prevention, followed by 20% who stated avoidance of contact with wild animals. Other answers that are not adequate measures of rabies prevention included: medication, not stressing the animals and taking care of the animals’ food, water and hygiene (Figure 5).

### 3.3. Attitudes and Practices

Almost all the families kept domestic animals, mainly dogs and cats. Most of them had vaccinated their companion animals (dogs and cats), but much less often their production animals, such as pigs, cattle and sheep (Table 4).

**Figure 6:**
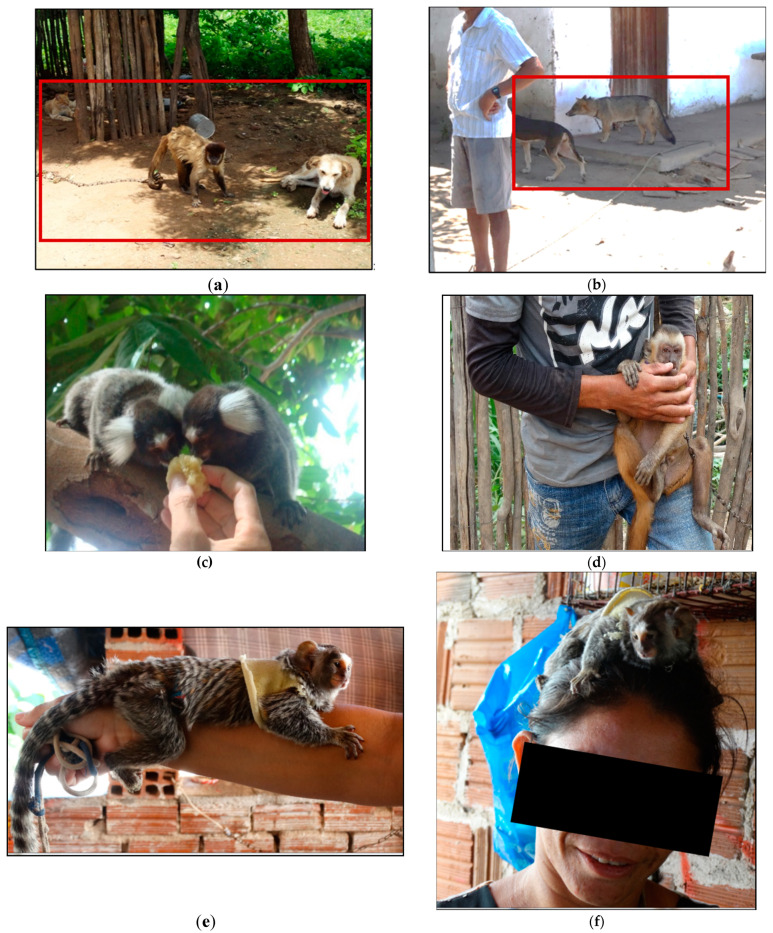

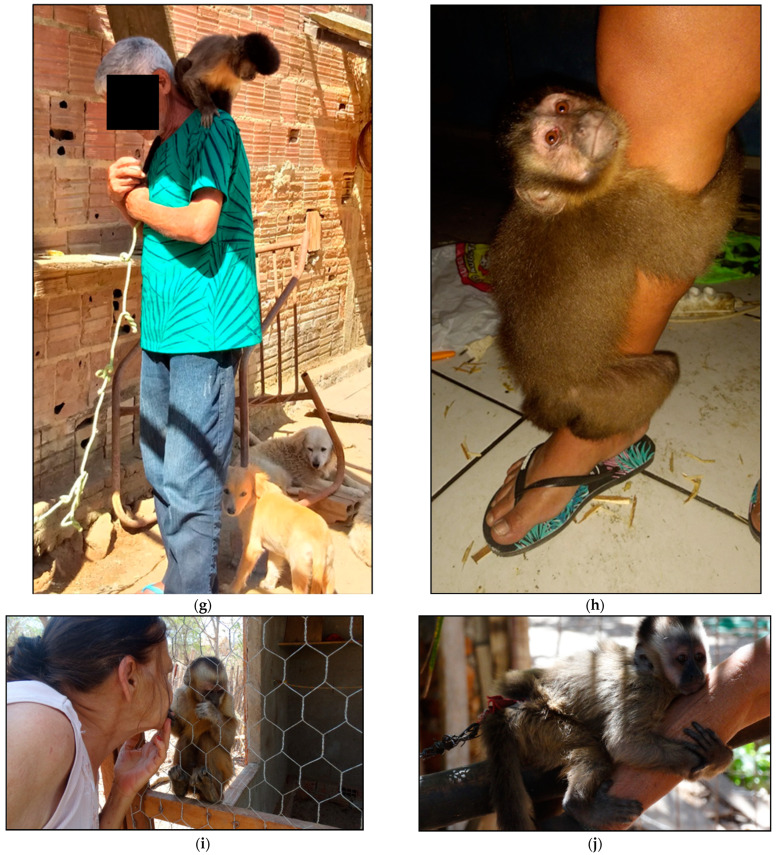
Examples of contact of wild animals with domestic animals and the population: (**a**) monkey living in the same environment as domestic dogs and cats; (**b**) domestic dog (*Canis familiaris)* and domesticated wild canid (*Cerdocyon thous*) living in the same environment; (**c**) human contact with marmosets in backyard; (**d**) human contact with monkey in backyard; (**e**,**f**) human contact with marmoset. (**g**–**j**) nail monkey breeders in contact with the animals.

Most families reported that free-living wild animals appeared on their properties, mostly marmosets and wild canids (*Cerdocyon thous*), and that these animals had contact with their domestic animals, especially marmosets with dogs and cats, and wild canids with humans and monkeys (Figure 6).

The animals appeared frequently in the backyards and on a daily basis (Figure 6, Table 5). The respondents also reported that animals, mostly monkeys (*Sapajus libidinosus*), marmosets (*Callithrix jacchus*) and wild canids (*C. thous*), had attacked domestic dogs and humans (Table 5).

**Figure 7:**
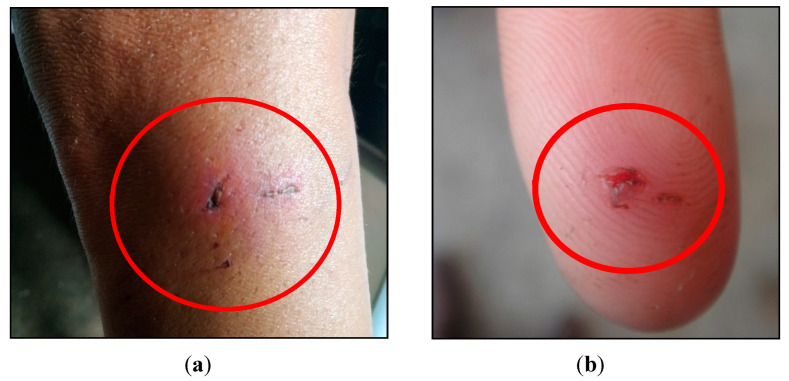
Examples of aggressions by wild animals to humans: (**a**) bite by a capuchin monkey at the leg of a person; (**b**) marmoset aggression on the index finger of a person.

About 73% said they had raised or currently raised wild animals as pets. These were mostly capuchin monkeys and marmosets. More than half stated that there had been aggressions to domestic animals and humans, with the majority of aggressions by capuchin monkeys and marmosets to humans (Figure 7), and about 62% of the humans attacked did not receive any anti-rabies post-exposure prophylaxis (Table 6).

After environmental education performed within the realm of data collection, 70% of the families that had animals in captivity decided to voluntarily surrender the animals to the competent bodies for rehabilitation and subsequent release into their natural habitats. Most of these were capuchin monkeys and marmosets.

## 4. Discussion

This is the first KAP study on sylvatic rabies in Brazil. Our data indicate some general knowledge about rabies and virus transmission in the studied high-risk population, but also incomplete knowledge, especially on the clinical signs of rabies in domestic and wild animals, preventive rabies management for animals and on the species that are important transmitters.

The results are a matter of concern since we have recently shown that, in Ceará State, rabies virus transmission dynamics have undergone important changes, with a shift from dog-mediated transmission to transmission by sylvatic populations [5,6]. In fact, previous studies from Ceará indicated that insufficient knowledge of populations living in high-risk areas was related to inadequate prevention measures after aggression by wild animals [6,12]. From 1990 to 2016, 19 cases of human rabies transmitted by marmosets were reported in Brazil, with the highest numbers of cases in Ceará and Piauí States [14]. Consequently, sylvatic rabies has been the focus of the rabies control programs, including information and education campaigns emphasizing the risk of wild animals transmitting rabies virus to humans.

In Brazil, keeping wild animals is prohibited by law, but about ¾ of the interviewed families in our study had raised wild animals as companion animals (mainly capuchin monkeys), and also the vast majority kept domestic animals (mostly dogs and cats) that interacted with sylvatic populations (in the wild and in captivity). On the other hand, domestic animals usually live intensively close to their owners, favoring the occurrence of aggressions. Thus, there is an increased risk of rabies virus transmission between wild animals, domestic animals and, consequently, humans. Most families were not fully aware about the legislation and the health risk of keeping wild animals and handed over their wild animals to the respective authorities after being informed about the rabies virus transmission risk. This indicates a high effectiveness of the focused information and education campaigns.

The common coexistence of domestic and wild animals as evidenced in this study and the circulation of rabies virus variants maintained and transmitted by wild animals (marmosets and wild canids) in the region further strengthen the importance of anti-rabies vaccinations of dogs and cats. Increased human/animal and domestic animal/wild animal interaction may also have been caused by the human invasion into former sylvatic areas, agricultural intensification and loss of natural habitats.

In the last five years, a large number of rabies virus-infected bats were identified in urban areas in Ceará [5]. Of the 21 registered human rabies cases in Brazil from 2016 to 2019, three (14.3%) were due to exposure by domestic cats, with the involvement of the variant of the hematophagous bat *Desmodus rotundus* [15]. This spillover from wild to domestic animals is of major concern considering the risk of unvaccinated domestic animals—only 50% of cats are vaccinated—coming into contact with these species. A spillover has been observed in Brazil since 2016, with the isolation of a virus variant from hematophagous bats and wild canids in domestic dogs and cats [16,17].

In contrast to other settings, such as in Kigali (Rwanda), where only 21% had vaccinated their dogs [18], a relatively high number of companion animals (especially dogs) have been vaccinated in Ceará State as a result of intensive campaigns for many years, but the production animals have been vaccinated to a much lesser extent. In several countries, mainly in Europe and North America, wild animals, such as foxes, have been vaccinated using vaccine-containing baits within the realm of eliminating wildlife-mediated rabies [19,20]. Although a study evidenced the production of antibodies against rabies after the use of a NIL-2 cell culture vaccine applied to marmosets (*C. jacchus*) in Ceará State [21], the practice of vaccinating wild animals is not yet recommended by the Brazilian Ministry of Health. In Brazil, vaccination of wild animals is not available as a control strategy, and additional laboratory and field studies are needed to assess if this approach is feasible within the northeast Brazilian setting.

Our study revealed that more than 30% of the people attacked by wild animals had not received any type of post-exposure prophylaxis, possibly because they were unaware of the transmission risk and the importance of post-exposure prophylaxis. A recent study has evidenced that all six human rabies cases that occurred during the last years in Ceará State in the period 2004–2021 were related to sylvatic rabies viral strains (five infections transmitted by wild animals, one by a domestic dog), and that only one patient had presented at a primary healthcare center before the onset of symptoms or clinical signs [12]. Another study from Fortaleza, the capital of Ceará State, evidenced that more than half of the people who kept marmosets as pets had been attacked by them at least once, and that about ¾ of these did not seek any health care center for receiving post-exposure prophylaxis; 58% did not know about the risk of transmission from the attack [22]. In general, respondents in rural areas had better knowledge on rabies virus transmission, symptoms, treatment and prevention than those residing in urban areas.

The situation is similar in other countries and settings. For example, in Pakistan, only 40% sought medical attendance after a dog bite [8]. Similar to our results, several studies from different settings in Grenada, Pakistan, Ghana and Ethiopia evidenced that rabies was known by the majority but that knowledge on transmission was insufficient, with only 40–60% mentioning animal bites as the principal way of transmission [8,18,23,24,25,26]. Only two respondents in our study knew about the importance of washing the wound with soap and water after a bite; a similar finding was reported in the Rwandan study [18]. All this evidence shows clearly that, in addition to the focus on wildlife-mediated rabies, information and education campaigns should consider insufficient knowledge of the population on transmission, immediate prevention measures and the importance of seeking healthcare after aggression by any animal (domestic or wild). In addition, access to the health system is an important factor and may have impacted the likelihood to seek healthcare in these different settings.

The One Health approach has been considered to be highly effective in controlling zoonotic diseases at the community level [27]. Within this realm, the Ceará State rabies control program has applied an integrated approach to rabies prevention and control, emphasizing environmental education, wild animal husbandry, legislation, zoonosis risk and animal welfare [13]. A multidisciplinary team has been assigned to address the different pillars of the control program. This team includes, for example, veterinarians, nurses, pharmacists, community health agents and information and education specialists [12]. These professionals are vaccinated, and their rabies-specific antibodies are assessed annually.

Our study is subject to limitations, such as the difficulty in identifying the families that had wild animals in captivity because most of them were afraid of being denounced and punished by environmental authorities. Given this context, the participants were included in collaboration with local community health agents and not recruited at random. As this purposive sampling procedure has been applied to include respondents of a specific high-risk group, the population is not representative of the general population. However, this study was not meant to include a representative sample of the state’s population but to draw a picture of the knowledge, attitudes and practices of populations at a high risk for acquiring rabies, with a focus on sylvatic populations. Another difficulty was the access to the homes of families that lived in rural areas where the largest number of families were registered, and the interruption of the visits due to the COVID-19 pandemic. Thus, the interpretation of the results regarding the external validity should be undertaken with care.

In conclusion, our study shows that there are important knowledge gaps within a high-risk population with contact to sylvatic populations, with inadequate practices regarding keeping wild animals and measures taken after animal aggressions. There is a need for strengthening and improving the sylvatic rabies surveillance and information and education programs to improve knowledge regarding virus transmission and preventive measures. Health education in high-risk communities should focus on sylvatic rabies, vaccination of domestic and farm animals and post-exposure prophylaxis.

## Figures and Tables

**Table 1:** Variables and questions on knowledge, attitudes and practices regarding rabies awareness and prevention, with focus on wildlife rabies, Ceará, Brazil.

Block 1	Socio-Demographic Characteristics
	Age, profession, education, municipality and area (urban/rural) of residence, number and age of inhabitants of the residence.
Block 2	Knowledge, attitudes and practices about the disease
	Have you ever heard about rabies?
	Which animal species can acquire the disease?
	What is the mode of transmission?
	What are the symptoms and signs?
	How do you prevent transmission?
	Do you know someone who has been bitten or scratched by animals (mammals) in the past?
Block 3	Presence of domestic animals, prophylaxis and animal contact
	Do you have domestic animals? Which species?
	Did the animals receive anti-rabies vaccination?
Block 4	Contact with wild animals
	Do wild animals appear and freely roam around on your compound? Which species?
	Do these wild animals have contact with domestic animals? What species have contact? Where and how often?
	Did you ever observe aggression of wild animals against domestic animals?
Block 5	Wildlife breeding
	Do you own or did you ever keep any wild animal?
	Did you ever have direct contact with wild animals? Which animals?
	Has there been any aggression against humans or domestic animals?

**Table 2:** Characteristics of study participants, Ceará, Brazil, 2019 (*n* = 113).

Variables	*n*	%
Sex		
Female	71	62.8
Male	42	37.1
Age group (years)		
18–39	26	23.0
40–59	48	42.5
>60	39	34.5
Education * (*n* = 104)		
Illiterate	22	19.5
Elementary	60	53.1
Primary school completed	17	15.0
Secondary/high school completed	5	4.4
Profession * (*n* = 109)		
Subsistence farmer	52	46.0
Employed	23	20.3
Home-maker	22	19.1
Retired	7	6.1
Informal worker	6	5.3
Student	1	0.9
Residential area		
Rural	61	54.0
Urban	52	46.0

* information not available in all cases.

**Table 3:** Knowledge about rabies, total and stratified by residential area, Ceará, Brazil (*n* = 113).

Questions	Total *n* (%)	Rural Area *n* (%)	Urban Area *n* (%)
Have you ever heard about the disease?			
Yes	104 (92.0)	59 (96.7)	45 (86.5)
No	9 (8.0)	2 (3.3)	7 (13.5)
Do you know which animal species can have the disease?			
Yes	90 (79.6)	52 (85.2)	38 (73.1)
No	23 (20.4)	9 (14.8)	14 (26.9)
Do you know how it is transmitted?			
Yes	70 (62.0)	43 (70.5)	27 (51.9)
No	43 (38.0)	18 (29.5)	25 (48.1)
Do you know what the symptoms are?			
Yes	75 (66.4)	46 (75.4)	29 (55.8)
No	38 (33.6)	15 (24.6)	23 (44.2)
Do you know how to prevent it?			
Yes	76 (67.3)	46 (75.4)	30 (57.7)
No	37 (32.7)	15 (24.6)	22 (42.3)
Do you know anyone who has been bitten or scratched by animals (mammals) (*n* = 79) *			
Yes	18 (22.8)	13 (29.6)	5 (14.3)
No	61 (77.2)	31 (70.4)	30 (85.7)
If yes, was there anti-rabies management? (*n* = 18)			
Yes	11 (61.1)	8 (61.5)	3 (60.0)
No	7 (38.9)	5 (38.5)	2 (40.0)

* In 34 of the interviewees, this question was not asked. See Material and Methods.

**Table 4:** Presence of domestic animals in the households and vaccination status, Ceará, Brazil, 2021 (*n* = 113).

Questions	*n*	%
Do you have domestic animals?		
Yes	106	93.8
No	7	6.2
Which domestic animals do you have? (*n* = 106)		
Domestic dog	85	80.2
Cat	65	61.3
Pig	30	28.3
Cattle	26	24.5
Sheep	21	19.8
Horse	18	17.0
Goat	18	17.0
Chicken	12	11.3
Donkey	3	2.8
Rabbit	1	0.9
Have the animals been vaccinated against rabies? (*n* = 105)		
Yes	90	85.7
No	15	14.3
Which animals have been vaccinated against rabies?		
Domestic dog	77/85	90.6
Cat	53/65	81.5
Sheep	10/21	47.6
Cattle	7/26	26.9
Goat	6/18	33.3
Pig	6/30	20.0
Horse	0/18	0
Rabbit	0/1	0
Donkey	0/3	0

**Table 5:** Presence of free-living wild animals on respondents’ properties and contacts between wild and domestic animals, Ceará, Brazil, 2019 (*n* = 113). Domestic dogs (*Canis familiaris*) include both owned dogs and feral dogs.

	*n*	%
Do free-living wild animals appear on your property?		
Yes	76	67.3
No	37	32.7
Which? (*n* = 76)		
Marmoset (*Callithrix jacchus*)	54	71.0
Wild canid (*Cerdocyon thous*)	22	28.9
Raccoon (*Procyon cancrivorus*)	17	22.4
Bat (Chiroptera)	13	17.1
Capuchin monkey (*Sapajus libidinosus*)	11	14.5
Wild cat (*Leopardus tigrinus*)	6	7.9
Jaguar (*Panthera onca*)	1	1.3
Where do the wild animals appear? (*n* = 73) *		
Backyard	42	57.5
Near the residence	23	31.5
Inside the residence	8	10.9
How often do they appear? (*n* = 71) *		
Daily	35	49.3
Weekly	13	18.3
Monthly	3	4.2
Annual	2	2.8
Sporadic	16	22.5
Did not know	2	2.8
Contact of wild species with domestics and humans? (*n* = 77)		
Yes	27	35.1
No	50	64.9
With which species? (*n* = 27)		
Marmoset with domestic dog	6	22.2
Marmoset with cat	6	22.2
Marmoset with human	3	11.1
Marmoset with monkeys	3	11.1
Capuchin monkey with domestic dog	3	11.1
Capuchin monkey with cat	2	7.4
Domestic dog with monkey	2	7.4
Bat with horse	1	3.7
Capuchin monkey with human	1	3.7
Aggression of wild animals on domestic domestic animals and humans? (*n* = 71) *		
Yes	13	18.3
No	58	81.7
Among which species? (*n* = 13)		
Wild canid to chicken	4	30.8
Capuchin monkey to human	2	15.4
Marmoset to human	1	7.7
Marmoset to chicken	1	7.7
Capuchin monkey to domestic dog	1	7.7
Skunk to domestic dog	1	7.7
Wild canid to human	1	7.7
Capuchin monkey to chicken	1	7.7
Bat to horse	1	7.7

* Data not available in all cases.

**Table 6:** Wild animals kept as companion animals, aggression and the type of contact between humans and animals on the respondents’ properties, Ceará, Brazil, 2019 (*n* = 113).

Questions	*n*	%
Do you own or have you ever owned a wild animal?		
Yes	83	73.4
No	30	26.6
How many animals? (*n* = 83)		
1	65	78.3
2	7	8.4
3	10	12.1
4	1	1.2
Which animals? (*n* = 83)		
Capuchin monkey	66	79.5
Marmoset	20	24.1
Wild canids	3	3.6
Maracaja cat (*Leopardus wiedii*)	2	2.4
Raccoon	1	1.2
Red deer	1	1.2
Ferret	1	1.2
Did wild animals have contact with humans or domestic animals in the household? (*n* = 68) *		
Yes	63	92.6
No	5	7.4
Which species? (*n* = 63)		
Monkey and human	54	85.7
Marmoset and human	7	11.1
Capuchin monkey and cat	5	7.9
Capuchin monkey and domestic dog	3	4.8
Wild canids and human	1	1.6
Deer and human	1	1.6
Capuchin monkey and goat	1	1.6
Capuchin monkey and pig	1	1.6
Marmoset and domestic dog	1	1.6
Marmoset and cat	1	1.6
Raccoon and human	1	1.6
Was there any aggression towards humans or domestic animals? (*n* = 83)		
Yes	45	54.2
No	38	45.8
Was there anti-rabies “treatment” (=post-exposure prophylaxis) of people after being attacked? (*n* = 45)		
Yes	15	33.3
No	28	62.2
Do not know	2	4.4

* *n* = 15 (18.1%) did not answer or left this question blank.

## Data Availability

The data presented in this study are available on reasonable request from the corresponding author.
